# KLOTHO-VS heterozygosity, α-klotho protein levels and cognitive performance in Alzheimer’s disease

**DOI:** 10.1186/s13195-025-01878-5

**Published:** 2025-10-29

**Authors:** Alzbeta Katonova, Ross Andel, Vanesa Jurasova, Katerina Veverova, Sarka Borovska, Hana Horakova, Tereza Kolarova, Vaclav Matoska, Martin Vyhnalek, Jakub Hort

**Affiliations:** 1 Memory Clinic, Department of Neurology, Second Faculty of Medicine, Charles University and Motol University Hospital, Prague, Czech Republic; 2 Edson College of Nursing and Health Innovation, Arizona State University, Phoenix, AZ USA; 3 Department of Clinical Biochemistry, Hematology and Immunology, Homolka Hospital, Prague, Czech Republic

**Keywords:** Alzheimer’s disease, α-Klotho, *KLOTHO*-VS heterozygosity, APOE, Memory, Cognition

## Abstract

**Background:**

*KLOTHO*-VS (*KL*-VS) heterozygosity, a variant of the *KLOTHO* gene, and its encoded protein, α-Klotho, are associated with brain health and show neuroprotective potential against Alzheimer’s disease (AD). We aimed to assess whether *KL*-VS heterozygosity, cerebrospinal fluid (CSF) and serum soluble α-Klotho (sαKl) levels, would be associated with a lower likelihood of AD and better performance on memory and other cognitive domains in individuals with AD dementia, amnestic mild cognitive impairment (aMCI) due to AD, and cognitively unimpaired controls.

**Methods:**

In this cross-sectional study, we analyzed two partially overlapping subsamples derived from 296 participants from the Czech Brain Aging Study. The first subsample included 196 participants with *KL*-VS haplotype data: 71 with AD dementia, 84 with aMCI due to AD, and 41 cognitively unimpaired controls. The second subsample included 147 participants with CSF and/or serum sαKl measurements, including 58 with AD dementia, 59 with aMCI due to AD, and 30 cognitively unimpaired controls. Diagnoses of aMCI and AD dementia were confirmed by positive CSF biomarkers and/or amyloid PET imaging. Logistic regression assessed how *KL*-VS heterozygosity influenced the odds of aMCI or dementia due to AD. Linear regression investigated associations between cognitive performance and either *KL*-VS heterozygosity or CSF/serum sαKl levels. Analysis of variance and analysis of covariance with post-hoc tests were used to compare sαKl levels across study groups.

**Results:**

*KL*-VS heterozygosity carriers showed a consistent trend towards lower odds of being classified with aMCI and dementia due to AD, with similar patterns in both Apolipoprotein E ε4 (*APOE* ε4) allele carriers and non-carriers, although none of the associations reached statistical significance despite moderate (rather than small) effect sizes. Among individuals with aMCI due to AD, *KL*-VS heterozygotes displayed better memory performance (β = 0.61, *p* = .008), particularly those who also carried the *APOE* ε4 allele (β = 0.64, *p* = .042). Results with other cognitive domains were non-significant. No significant differences in sαKl levels were found between study groups, and soluble α-Klotho levels did not associate with memory performance.

**Conclusions:**

*KL*-VS heterozygosity may be linked to lower likelihood of classification as aMCI or dementia due to AD, and its association with memory might be specific to the aMCI stage of AD and modulated by *APOE* ε4 status.

**Supplementary Information:**

The online version contains supplementary material available at 10.1186/s13195-025-01878-5.

## Background

α-Klotho, encoded by the *KLOTHO* gene, is a single-pass transmembrane protein primarily expressed in the brain’s choroid plexus and the kidney’s distal tubule cells [1]. A soluble form of α-Klotho (sαKl) is produced through proteolytic cleavage from membrane-bound α-Klotho and released into the bloodstream or cerebrospinal fluid (CSF), where it may act as a hormone exerting multiple systemic biological actions on cells or tissues that do not express α-Klotho [2].

α-Klotho’s role in the aging process was first identified through a study on *KLOTHO* knockout mice, whereby poor *KLOTHO* gene expression was shown to significantly reduce life expectancy through mechanisms similar to those seen in human aging [3]. Later research focusing on the central nervous system of the transgenic mice revealed that they also exhibited age-related neurodegenerative changes, including synaptic loss, neuronal degeneration, and cognitive impairment [4, 5]. On the other hand, overexpression of α-Klotho in mice was found to extend lifespan, improve learning, memory, synaptic plasticity and reduce amyloid-β (Aβ) burden [6–9]. While transgenic mice models provided compelling evidence for α-Klotho’s neuroprotective potential, human studies offer a crucial bridge to understanding its in vivo relevance in neurodegenerative diseases.

*KLOTHO*-VS heterozygosity (*KL*-VSHET), a functional genotype of the *KLOTHO* gene, has been associated with various beneficial effects, particularly in the context of AD and cognitive function [6, 10–16]. Specifically, having the *KL*-VSHET haplotype has been shown to significantly decrease the risk of AD, although the effect appears to be restricted to carriers of the Apolipoprotein E ε4 allele (*APOE* ε4) [11]. This finding may be a function of the ability of the *KL*-VSHET to attenuate the adverse effects of *APOE* ε4 on Aβ accumulation and tau burden [10–12, 14, 15, 17]. Additionally, evidence suggests that *KL*-VSHET is associated with better memory even in Aβ driven pathology, likely due to lowering the levels of tau protein [14]. One of the primary mechanisms by which *KL*-VSHET may confer resilience against AD is by increasing the levels of both CSF and serum sαKl [6, 18, 19].

While the promising associations between higher sαKl levels and better cognitive performance are evident in normal aging [20, 21], its role in AD remains unclear. Studies in AD patients link lower CSF sαKl to worse AD pathology and cognitive function [18, 22, 23]. In contrast, plasma sαKl shows inconsistent results, with some studies finding no difference between AD patients and controls [18, 24] and others reporting higher levels in AD patients [25]. This study extends prior work by assessing domain-specific cognition in biomarker-confirmed AD groups and evaluating both sαKl protein levels and *KL*-VS haplotype.

Based on previous research, we investigated whether *KL*-VSHET would be associated with a lower likelihood of aMCI or dementia due to AD compared to controls in *APOE* ε4 carriers vs. non-carriers. We expected positive results in *APOE* ε4 carriers but not in *APOE* ε4 non-carriers. We also tested whether *KL*-VSHET carriers would have better cognitive performance than those who were *KL*-VS non-carriers (*KL*-VSNC). Given prior evidence suggesting that *KL*-VSHET mitigates AD pathology predominantly in *APOE ε4* carriers, we hypothesized that associations between *KL*-VSHET and both cognitive performance and likelihood of classification as AD would be stronger in individuals carrying the *APOE* ε4 allele. This expectation was grounded in multiple large-scale studies demonstrating that *KL*-VSHET attenuates *APOE ε4*–associated Aβ accumulation and cognitive decline in cognitively normal individuals at genetic risk for AD​​​. These protective associations appear to be *APOE* ε4-dependent, with most studies reporting little to no benefit of *KL*-VSHET in *APOE* ε4 non-carriers​​ [10, 12, 17]. Our study sought to extend this line of research by evaluating whether such *APOE* ε4-specific effects would persist across clinically defined stages of AD. Additionally, we sought to compare sαKl concentrations in CSF and in serum across patients with AD dementia, aMCI due to AD, and cognitively unimpaired individuals. We hypothesized that patients with AD dementia and with aMCI due to AD would exhibit lower levels of sαKl in CSF compared to cognitively unimpaired controls. Finally, we investigated whether CSF and serum sαKl would be associated with better cognitive performance.

## Methods

### Participants

Participants were drawn from the Czech Brain Aging Study cohort [26] at the Memory Clinic of Charles University/Motol University Hospital, and the Department of Neurology, Motol University Hospital in Prague, Czech Republic. All Czech Brain Aging Study participants are subjected to clinical and laboratory evaluations within three months of the initial visit, including routine blood tests, comprehensive neuropsychological assessment, and brain magnetic resonance imaging (MRI; 1.5 or 3 T with MP RAGE sequences). All participants involved in this study signed written informed consent approved by the Motol University Hospital ethics committee. All participants included in the study were White and of Czech nationality.

### Inclusion/exclusion criteria

To be included in the present study, participants were required to meet criteria for one of three diagnostic groups—AD dementia, aMCI due to AD, or cognitively unimpaired controls—and to have either sαKl protein levels measured in CSF and/or serum or genotyping data available for the *KL*-VS haplotype. Participants were excluded if they had pre-existing neurological or psychiatric conditions that could impair cognitive function, including Parkinson disease, Lewy body dementia, frontotemporal lobar degeneration, psychosis, substance abuse, depression (≥ 6 points on the 15-item Geriatric Depression Scale) [27], stroke, traumatic brain injury, or multiple sclerosis. Additionally, patients with severe white matter vascular lesions on MRI (Fazekas score > 2 points) [28] were excluded. Participants suffering from renal failure (defined as a GFR min/1.73 m² or chronic kidney damage) were also excluded since kidneys are a major site of α-Klotho production. Participants were also excluded from the study sample due to *KL*-VS homozygosity, as this genotype is rare in the general population and would not permit meaningful statistical analysis.

### Study sample

**Fig. 1:**
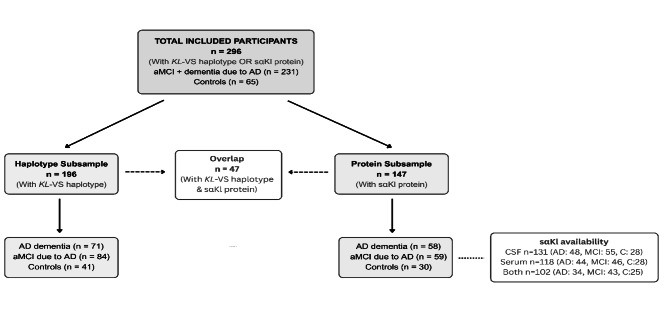
Flow diagram illustrating the composition of the study sample. A total of 296 participants were included based on the availability of either *KL-VS* haplotype genotyping or sαKl protein data. Two partially overlapping subsamples were derived: the Haplotype Subsample (*n* = 196), consisting of participants with *KL-VS* haplotype data, and the Protein Subsample (*n* = 147), consisting of participants with CSF and/or serum sαKl protein concentrations. A total of 47 participants were included in both subsamples. Each subsample was categorized by diagnostic group: AD dementia, aMCI due to AD, and cognitively unimpaired controls. Diagnostic group assignment required biomarker confirmation. All participants with AD dementia or aMCI due to AD were CSF and/or PET positive; all cognitively unimpaired controls were CSF and/or PET negative

An overview of the composition of the two subsamples used in this study is shown in Fig. 1. Following application of these inclusion and exclusion criteria, a total of 296 participants had relevant data available and were included in the present study. Of the 296 participants, 231 were referred to the Memory Clinic by general practitioners, neurologists, or geriatricians based on cognitive difficulties reported by themselves or their informants. These participants comprise the diagnostic groups of AD dementia and aMCI due to AD used in both the haplotype and protein subsamples (see Fig. 1). The remaining 65 participants were cognitively unimpaired controls.

Based on available genetic and biomarker data, participants were divided into two partially overlapping analytic subsamples: one with available *KL*-VS haplotype (haplotype subsample), and one with available sαKl protein measurements in CSF and/or serum (protein subsample).

The haplotype subsample consisted of 196 participants and was used to examine the association between *KL*-VSHET and odds of AD as well as cognitive performance. It included 71 participants with AD dementia [29], 84 with aMCI due to AD [30], and 41 cognitively unimpaired controls. AD biomarker status in this subsample was determined using CSF (*n* = 129), Aβ PET imaging (*n* = 96), or both modalities (*n* = 29).

The protein subsample consisted of 147 participants and was used to investigate differences in sαKl levels across clinical groups and their associations with cognitive outcomes. It comprised 58 participants with AD dementia [29], 59 with aMCI due to AD [30], and 30 cognitively unimpaired controls. CSF sαKl levels were available for 131 participants (AD dementia *n* = 48, aMCI due to AD *n* = 55, controls *n* = 28), and serum sαKl levels were available for 118 participants (AD dementia *n* = 44, aMCI due to AD *n* = 46, controls *n* = 28). Both CSF and serum sαKl measurements were available for 102 participants (AD dementia *n* = 34, aMCI due to AD *n* = 43, controls *n* = 25). AD biomarker status in this subsample was determined using CSF (*n* = 147), Aβ PET imaging (*n* = 42), or both modalities (*n* = 42).

There was partial overlap between the two subsamples, with 47 participants contributing data to both analyses (AD dementia *n* = 21, aMCI due to AD *n* = 18, controls *n* = 8).

### Diagnostic information

Participants diagnosed with AD dementia met the NIA-AA 2011 criteria for dementia due to AD [29] based on progressive decline in at least two cognitive domains (i.e. ≥1.5 standard deviations (SD) lower memory test score than the age- and education-adjusted norms as well as similarly low score in at least one non-memory cognitive test), significant impairment in the ability to perform daily activities, and biomarker evidence of AD pathophysiology. Biomarker confirmation was established via either lumbar puncture or Aβ positron emission tomography (PET) imaging using 18 F-flutemetamol. Positive biomarker status was defined as CSF Aβ42 < 620 pg/mL and phosphorylated tau at amino acid 181 > 61 pg/mL and/or a positive Aβ PET scan.

Participants diagnosed with aMCI due to AD met the NIA-AA 2011 criteria for MCI [30] based on subjective reports of memory decline compared to their prior level, objective evidence of memory impairment (i.e. ≥1.5 SDs lower score than the age- and education-adjusted norms in any memory test), preserved independence in daily activities, and the absence of dementia. As with the AD dementia group, aMCI participants were also required to have biomarker confirmation of AD pathology, based on either CSF analysis (Aβ42 < 620 pg/mL and phosphorylated tau > 61 pg/mL) or a positive Aβ PET scan.

Cognitively unimpaired individuals were required to exhibit normal cognitive function (scores > 1.5 SD above age- and education-adjusted norms on all cognitive tests) and normal AD biomarkers in CSF and/or PET. These participants were recruited from two sources:

- among individuals initially referred to the Department of Neurology, Motol University Hospital, for lumbar puncture to rule out central nervous system inflammation. After normal CSF analysis, absence of systemic inflammation, and no evidence of hippocampal atrophy on MRI, these individuals were enrolled into the Czech Brain Aging Study as cognitively unimpaired controls.
- among Czech Brain Aging Study patients followed for subjective cognitive decline. Although they reported cognitive difficulties that led them to seek medical evaluation, they showed unimpaired activities of daily living and normal cognitive function [31]. MRI findings revealed no hippocampal atrophy, and AD biomarkers in CSF or PET were within normal ranges.

### Genotyping

*APOE* and *KLOTHO* genotypes were determined at the Department of Clinical Biochemistry, Hematology and Immunology, Homolka Hospital, Prague, Czech Republic.

DNA isolation was performed by Zybio Nucleic extraction kit WB-B from whole blood samples according to manufacturer’s protocol (Zybio, Chongqing, China).

*APOE* genotyping was performed according to IdahoTech protocol (Luna Probes Genotyping Apolipoprotein [ApoE] Multiplexed Assay) for high-resolution melting analysis (HRM) [32, 33].

The analysis of *KL*-VS haplotype was done also by HRM analysis of single nucleotide polymorphism rs9536314 (G/T). The reaction was performed with LightScanner Master mix (BioFire Diagnostics, SLC., USA) according to manufacturer’s PCR reaction conditions with forward and reverse primers: KL1F 5 ´- ATAACCTTTCATCTATTCTGC-3´; KL1R 5 ´- AAGTCAGCAGTTCCTTTG-3´; Temperature profile was: 95 °C for 2 min followed by 40 cycles of 95 °C/30s; 63 °C/30s; 72 °C/30s; Melting 60–90 °C. HRM analysis was performed on LightScanner (IdahoTech).

### CSF and blood collection and processing

Whole blood was collected by venipuncture. Samples were allowed to clot for 15 min at room temperature before centrifugation at 1700 x g for 5 min at 20 °C. The resulting serum supernatant was aliquoted into 0.5 ml polypropylene tubes and stored at -80 °C until further analysis. CSF was obtained by lumbar puncture in a supine position at L3/L4 or L4/L5. Following collection in 8 ml polypropylene tubes, samples were gently mixed and centrifuged at 1700 x g for 5 min at 20 °C. The supernatant was then aliquoted into 0.5 ml polypropylene tubes and stored at -80 °C until analysis. Before analysis, serum and CSF samples were thawed at room temperature and vortexed for 15 s for thorough homogenization.

### Immunological assays

Protein levels of sαKl in serum and CSF were quantified using a commercially available enzyme-linked immunosorbent assay kit (Immuno-Biological Laboratories Co Ltd, Japan; cat. no. JP27998) following the manufacturer’s instructions. Serum and CSF samples were measured in duplicate and were analyzed undiluted. At the end of the assay, absorbances were read at 450 nm using a microplate reader (Dynex Technologies, Virginia, USA), and the protein concentration was calculated by comparison with a standard curve. The intra-assay coefficient of variance (CV%) was < 3%, and the inter-assay CV was < 8%.

### Neuropsychological assessment

The neuropsychological test battery included the following measures: Mini-Mental State Examination (MMSE) [34]; Forward and Backward Digit Span subtests (DS-F, DS-B, respectively), an adaptation from the Uniform Data Set (UDS-cz 2.0) [35]; Trail Making Test (TMT) A and B [36]; Logical Memory (LM) immediate and delayed recall, an adaptation from the UDS-cz 2.0 [35]; Boston Naming Test (BNT-30), 30 odd-items version [37]; semantic verbal fluency (S-VF, animals) and phonemic verbal fluency (P-VF, Czech version with letters N, K, P) [38]; Rey-Osterrieth Complex Figure Task (ROCFT)—the copy condition [39]; and the Clock Drawing Test (CDT) [40].

### Statistical analyses

To evaluate between-group differences in age, years of education, and global cognitive functioning as assessed by MMSE, we used one-way analysis of variance (ANOVA) with Tukey’s Honestly Significant Differences (HSD) post hoc tests. To evaluate sex, *KL*-VSHET and *APOE* ε4 frequency differences across groups we used the χ2 test.

Normality was assessed through the inspection of histograms, skewness, and the Shapiro-Wilks test of normality. Serum sαKl had nonnormal distribution. Therefore, log transformation was applied.

Cognitive domains for the participants were expressed as composite domain z-scores, computed by standardizing the raw scores for each neuropsychological test to z-scores using the mean and SD for the entire sample and subsequently averaging these to create single composite scores for attention and working memory (DS-F, DS-B, TMT A), memory (LM immediate and delayed recall), executive function (TMT B, P-VF), language (S-VF, BNT-30), and visuospatial function (ROCFT, CDT). Scores of TMT A and B, and BNT-30 errors were reversed before transformation to z-scores, to express the values in the same direction as the other neuropsychological values. The maximum time for completion of the TMT A and B were 180s and 300s, respectively, and those who were unable to complete the tests were assigned a score of 181s and 301s, respectively.

To examine the relationship between *KL*-VS haplotype and the odds of aMCI or dementia due to AD compared to individuals who were cognitively unimpaired, we first performed unadjusted binary logistic regression analysis with study group (cognitively unimpaired vs. either aMCI or dementia due to AD) as the dependent variable and *KL*-VS haplotype as the independent variable, followed by adjusted binary logistic regression adjusted for age, sex, and years of education. Logistic regression was interpreted using odds ratios (OR) and 95% confidence intervals, which correspond to a two-tailed 0.05 significance level. OR > 1.0 signifies an increased odds and OR < 1.0 reduced odds. ORs were subsequently interpreted as Cohen’s d [41, 42]. Statistical power was calculated using G*Power software [43]. Power estimates were based on the observed effect sizes (ORs from logistic regression), sample sizes, and an alpha of 0.05 (two-tailed).

To assess the association of *KL*-VSHET with cognitive performance, we first used univariate linear regression with cognitive domain z-score as the dependent variable and *KL*-VS haplotype as the independent variable, followed by multiple linear regression adjusted for age, sex, and years of education.

Between-group differences in sαKl levels were evaluated using one-way ANOVA, followed by one-way analysis of covariance (ANCOVA) controlling for age and sex. Significant main effects were interpreted using the Tukey’s HSD post hoc test. Each model included the mean value of sαKl as the outcome and the study group as a between-subject factor.

To investigate the association between sαKl protein levels and memory performance, we first used univariate linear regression with cognitive domain z-score as the dependent variable and CSF or serum sαKl levels as the independent variable, followed by multiple linear regression adjusted for age, sex and years of education.

Analyses were performed using R statistical language environment [44]. A two-tailed *p*-value < 0.05 in all of the analyses was considered statistically significant.

## Results

### Demographic characteristics

Table 1 summarizes demographics and clinical characteristics of participants with available *KLOTHO* genotyping. Sex distribution was similar across groups, but participants with aMCI due to AD and AD dementia were significantly older (*p* < .001, *p* = .017, respectively) and had fewer years of education (*p* = .034, *p* = .008, respectively) compared to controls. Both AD groups carried the *APOE* ε4 allele at significantly higher rates (*p* = .008, *p* = .003, respectively) than controls. While *KL-*VSHET carrier proportions differed between aMCI due to AD and controls (*p* = .029), no significant difference existed between AD dementia and controls. Finally, both AD groups scored significantly lower on the MMSE (*p* < .001 for both) and all neuropsychological tests compared to controls.

**Table 1:** Characteristics of study participants with available *KL*-VS haplotype

	AD dementia patients (*n* = 71)	aMCI due to AD patients (*n* = 84)	Controls (*n* = 41)	*p*-value AD dementia patients vs. controls	p*-*value aMCI due to AD patients vs. controls	*p*-value aMCI due to AD vs. AD dementia patients
**Demographic characteristics**						
Female/Male	39/32	43/41	26/15	0.381	0.197	0.562
Age in years	71.6 ± 8.9	73.0 ± 8.0	67.1 ± 8.8	0.017	< 0.001	0.585
Education in years **	14.8 ± 2.8	15.1 ± 2.9	16.5 ± 2.8	0.008	0.034	0.751
*APOE* e4 carriers, n (%) *	45 (63.3)	49 (58.3)	13 (31.7)	0.003	0.008	0.401
*KL*VS-HET, n (%)	20 (28.2)	16 (19.0)	15 (36.6)	0.358	0.029	0.161
MMSE score ***	20.0 ± 4.2	25.6 ± 2.5	28.9 ± 1.1	< 0.001	< 0.001	< 0.001
**Neuropsychological tests**						
DS-F	7.2 ± 1.7	8.4 ± 2.0	9.6 ± 2.3	< 0.001	0.014	0,008
DS-N	3.9 ± 1.8	5.2 ± 1.8	6.9 ± 2.2	< 0.001	< 0.001	0,004
TMT A	102.2 ± 52.3	57.0 ± 32.9	38.0 ± 10.8	< 0.001	0.031	< 0.001
TMT B	269.2 ± 63.2	178.1 ± 87.9	86.2 ± 27.2	< 0.001	< 0.001	< 0.001
S-VF	11.4 ± 5.4	18.9 ± 5.8	25.4 ± 5.0	< 0.001	0,005	< 0.001
P-VF	25.8 ± 13.1	39.7 ± 14.4	48.9 ± 14.2	< 0.001	< 0.001	< 0.001
BNT	9.3 ± 5.5	5.3 ± 4.3	2.0 ± 1.7	< 0.001	< 0.001	< 0.001
CDT	10.1 ± 4.0	13.2 ± 3.1	15.3 ± 1.0	< 0.001	0.003	< 0.001
ROCF-C	18.6 ± 11.0	26.7 ± 5.4	30.6 ± 3.1	< 0.001	0.017	< 0.001
LM-IR	4.8 ± 3.7	10.4 ± 4.5	15.5 ± 5.1	< 0.001	< 0.001	< 0.001
LM-DR	1.7 ± 2.6	6.5 ± 6.0	14.8 ± 5.4	< 0.001	< 0.001	< 0.001

Values are presented as mean ± SD except for sex and *APOE ε 4*. *P* values are comparisons using ANOVA with Tukey’s HSD post hoc test for continuous variables and chi square test for categorical variables

Abbreviations: **aMCI**, amnestic mild cognitive impairment; **AD**, Alzheimer’s disease; **APOE**, apolipoprotein E; **MMSE**, Mini Mental State Examination; **DS-F**, Digit Span Forward; **DS-B**, Digit Span Backward; **TMT**, Trail Making Test; **P-VF**, phonemic verbal fluency (letters N, K, P); **S-VF**, semantic verbal fluency (animals); **BNT**, Boston Naming Test (30 odd-items version), number of errors; **CDT**, Clock Drawing Test; **ROCF-C**, Rey-Osterrieth Complex Figure, copy condition; **LM-IR**, Logical Memory Story I, immediate recall; **LM-DR**, Logical Memory Story I, delayed recall

* missing genotype data in *n* = 3 (1 AD-MCI, 2 controls) ** missing education data in *n* = 8 (6 AD-dementia, 2 AD-MCI) *** missing MMSE data in *n* = 25 (15 AD-dementia, 9 AD-MCI, 1 control)

Table 2 summarizes demographics and clinical characteristics of participants with available CSF or serum based sαKl factor. Compared to controls, the aMCI due to AD and AD dementia groups had significantly more males (*p* = .007, *p* = .013, respectively). Both AD groups were significantly older than controls (*p* < .001 for both). While the AD dementia group had fewer years of education than controls (*p* = .045), this difference wasn’t significant for aMCI due to AD (*p* = .226). As expected, *APOE* ε4 positivity was more frequent in both aMCI due to AD (*p* < .001) and AD dementia (*p* = .003) compared to controls. Finally, both AD groups scored significantly lower in all neuropsychological tests compared to controls.

**Table 2:** Characteristics of study participants with available CSF or serum based sαKl factor

	AD dementia patients (*n* = 58)	aMCI due to AD patients (*n* = 59)	Controls (*n* = 30)	*p*-value AD dementia patients vs. controls	*p*-value aMCI due to AD patients vs. controls	*p*-value aMCI due to AD vs. AD dementia patients
**Demographic characteristics**						
Female/Male	38/20	37/22	27/3	0.013	0.007	0.752
Age in years	74.7 ± 5.4	73.6 ± 4.9	63.8 ± 8.5	< 0.001	< 0.001	0.574
Education in years **	14.0 ± 2.9	14.5 ± 3.1	15.6 ± 2.9	0.045	0.226	0.603
*APOE* e4 carriers, n (%) *	36 (62.1)	45 (76.3)	6 (20.0)	0.003	< 0.001	0.252
MMSE score **	18.7 ± 4.5	25.1 ± 2.7	28.9 ± 1.3	< 0.001	< 0.001	< 0.001
**SαKl levels**						
CSF sαKl, pg/ml	1196.3 ± 262.4	1276.4 ± 227.0	1359.5 ± 208.1	0.028	0.364	0.217
Serum sαKl, pg/ml	962.8 ± 316.9	992.3 ± 392.6	1129.1 ± 459.0	0.173	0.293	0.927
**Neuropsychological tests**						
DS-F	7.3 ± 1.9	8.3 ± 2.0	9.4 ± 2.0	< 0.001	0.034	0.054
DS-B	3.9 ± 1.4	4.9 ± 1.5	6.8 ± 1.7	< 0.001	< 0.001	0.003
TMT A	111.2 ± 56.2	63.1 ± 34.1	36.7 ± 10.6	< 0.001	0.007	< 0.001
TMT B	280.2 ± 48.1	197.9 ± 87.5	108.5 ± 65.0	< 0.001	< 0.001	< 0.001
S-VF	11.2 ± 5.3	16.1 ± 5.0	27.1 ± 6.3	< 0.001	< 0.001	< 0.001
P-VF	24.1 ± 10.1	37.4 ± 12.5	50.5 ± 11.8	< 0.001	< 0.001	< 0.001
BNT	11.2 ± 4.8	6.8 ± 4.6	1.4 ± 1.7	< 0.001	< 0.001	< 0.001
CDT	9.5 ± 3.8	12.9 ± 2.1	15.4 ± 0.7	< 0.001	0.001	< 0.001
ROCF-C	19.7 ± 10.1	25.1 ± 6.8	30.0 ± 3.0	< 0.001	0.008	0.003
LM-IR	4.7 ± 3.4	8.3 ± 4.1	18.4 ± 4.0	< 0.001	< 0.001	< 0.001
LM-DR	1.4 ± 2.2	3.5 ± 5.8	16.4 ± 5.4	< 0.001	< 0.001	0.137

Values are presented as mean ± SD except for sex and *APOE ε4*. *P* values are comparisons using ANOVA with Tukey’s HSD post hoc test for continuous variables and chi square test for categorical variables

Abbreviations: **aMCI**, amnestic mild cognitive impairment; **AD**, Alzheimer’s disease; **APOE**, apolipoprotein E; **MMSE**, Mini Mental State Examination; **DS-F**, Digit Span Forward; **DS-B**, Digit Span Backward; **TMT**, Trail Making Test; **P-VF**, phonemic verbal fluency (letters N, K, P); **S-VF**, semantic verbal fluency (animals); **BNT**, Boston Naming Test (30 odd-items version), number of errors; **CDT**, Clock Drawing Test; **ROCF-C**, Rey-Osterrieth Complex Figure, copy condition; L**M-IR**, Logical Memory Story I, immediate recall; **LM-DR**, Logical Memory Story I, delayed recall

*missing genotype data in *n* = 16 (5 AD-dementia, 1 AD-MCI, 10 controls) ** missing education and MMSE data in *n* = 3 (3 AD-dementia)

Compared to men, women had significantly higher sαKl levels in both CSF (1291.8 ± 243.5 pg/mL vs. 1189.1 ± 230.6 pg/mL, *p* = .032) and serum (1059.2 ± 445.7 pg/mL vs. 915.8 ± 179.7 pg/mL, *p* = .014). There were no significant differences in CSF or serum sαKl levels based on *APOE* ε4 status. Both CSF and serum sαKl levels were weakly, negatively correlated with age in the entire study sample (*r*=-.28; *p* = .002, and *r*=-.26; *p* = .004, respectively). In addition, there was a weak positive correlation between CSF and serum sαKl in the entire sample (*r* = .21, *p* = .042).

### Association of -VS heterozygosity with AD diagnostic group classification

The ORs with 95% confidence intervals (CI) for each of the analyses are presented in Table 3. Carriers of the *KL*-VSHET haplotype had 61% lower odds of being classified as aMCI due to AD compared to cognitively unimpaired group (OR = 0.39, 95% CI 0.14–1.04, *p* = .061), although this association only trended towards significance, with a statistical power of 0.66 to detect an effect of this size. The magnitude of this association was similar for both *APOE* ε4 carriers (OR = 0.41, 95% CI 0.07–2.34, *p* = .306) and non-carriers (OR = 0.45, 95% CI 0.10–1.86, *p* = .274), but neither was statistically significant. The power to detect these effects was low (0.29 and 0.33, respectively).

**Table 3:** Logistic regression models showing the association between *KL*-VSHET and the odds of aMCI or dementia due to AD

	Model 1				Model 2			
**Groups compared**	OR	95% CI	*p*	Cohen’s d	OR	95% CI	*p*	Cohen’s d
Controls – MCI-AD	0,40	0.17–0.92	**0.032**	0,51	0,39	0.14–1.04	0.061	0,52
Controls – MCI-AD (*APOE*4+)	0,42	0.11–1.65	0.198	0,48	0,41	0.07–2.34	0.306	0,49
Controls – MCI-AD (*APOE*4-)	0,38	0.11–1.25	0.116	0,53	0,45	0.10–1.86	0.274	0,44
MCI-AD – AD dem	0,59	0.27–1.24	0.163	0,29	0,52	0.23–1.13	0.102	0,36
MCI-AD – AD dem (*APOE*4+)	0,72	0.27–1.89	0.509	0,18	0,59	0.20–1.63	0.309	0,29
MCI-AD – AD dem (*APOE*4-)	0,43	0.12–1.45	0.175	0,47	0,48	0.13–1.68	0.253	0,40
Controls – AD	0,52	0.24–1.10	0.081	0,36	0,59	0.26–1.36	0.205	0,29
Controls – AD (*APOE*4+)	0,50	0.15–1.78	0.258	0,38	0,50	0.12–2.08	0.321	0,38
Controls – AD (*APOE*4-)	0,57	0.21–1.59	0.271	0,31	0,72	0.22–2.36	0.579	0,18

Note. Model 1 is unadjusted, Model 2 is adjusted for age, sex, and education. AD refers to a combined group of participants with aMCI due to AD and AD dementia

Abbreviations: **OR**, odds ratio, **95% CI**, 95% confidence interval

*KL*-VSHET carriers displayed 48% lower odds of being among patients with AD dementia vs. with aMCI due to AD (OR = 0.52, 95% CI 0.23–1.13). While not statistically significant (*p* = .102), the analysis had moderate power (0.50) to detect an effect of this size. The ORs were similar regardless of *APOE* ε4 status, with 41% lower odds observed in *APOE* ε4 carriers (OR 0.59, 95% CI 0.20–1.63, *p* = .309) and 52% in non-carriers (OR 0.48, 95% CI 0.13–1.68, *p* = .253). However, both results lacked statistical significance, likely due to low power (0.24 and 0.27, respectively).

Finally, *KL*-VSHET was associated with 41% lower odds of being classified with either aMCI due to AD or AD dementia compared to being cognitively unimpaired (OR = 0.59, 95% CI 0.26–1.36, *p* = .205). The power to detect this effect size was low (0.32). The same model stratified by *APOE* ε4 status revealed that the association appeared stronger in *APOE* ε4 carriers (OR 0.50, 95% CI 0.12–2.08, *p* = .321) compared to non-carriers (OR 0.72, 95% CI 0.22–2.36, *p* = .579). However, neither association reached statistical significance, and both analyses had low power (0.22 and 0.11, respectively).

### Association of KL-VS heterozygosity with cognitive performance across study groups

*KL*-VSHET carriers in the aMCI due to AD group displayed significantly higher memory scores compared to *KL*-VSNC after controlling for age, sex, and years of education (β = 0.61, *p* = .008). After stratifying the aMCI due to AD group based on *APOE* ε4 status, this positive association remained significant only in the *APOE* ε4 carriers (β = 0.64, *p* = .042), and although it was still present, it was not significant in *APOE* ε4 non-carriers (β = 0.49, *p* = .136). Notably, this trend was not observed in the AD dementia or control groups, nor in the analysis of the entire sample. Additionally, no significant associations were found between *KL*-VSHET and any other cognitive domains in either the *APOE* ε4 carriers or *APOE* ε4 non-carriers or in any of the study groups (all *p* > .05).

The regression coefficients of each linear regression model showing *KL*-VSHET in relation to cognitive composite scores across study groups are presented in Supplementary Table 1, Additional File 1.

### Concentrations of CSF sαKl and serum sαKl across study groups

**Fig. 2:**
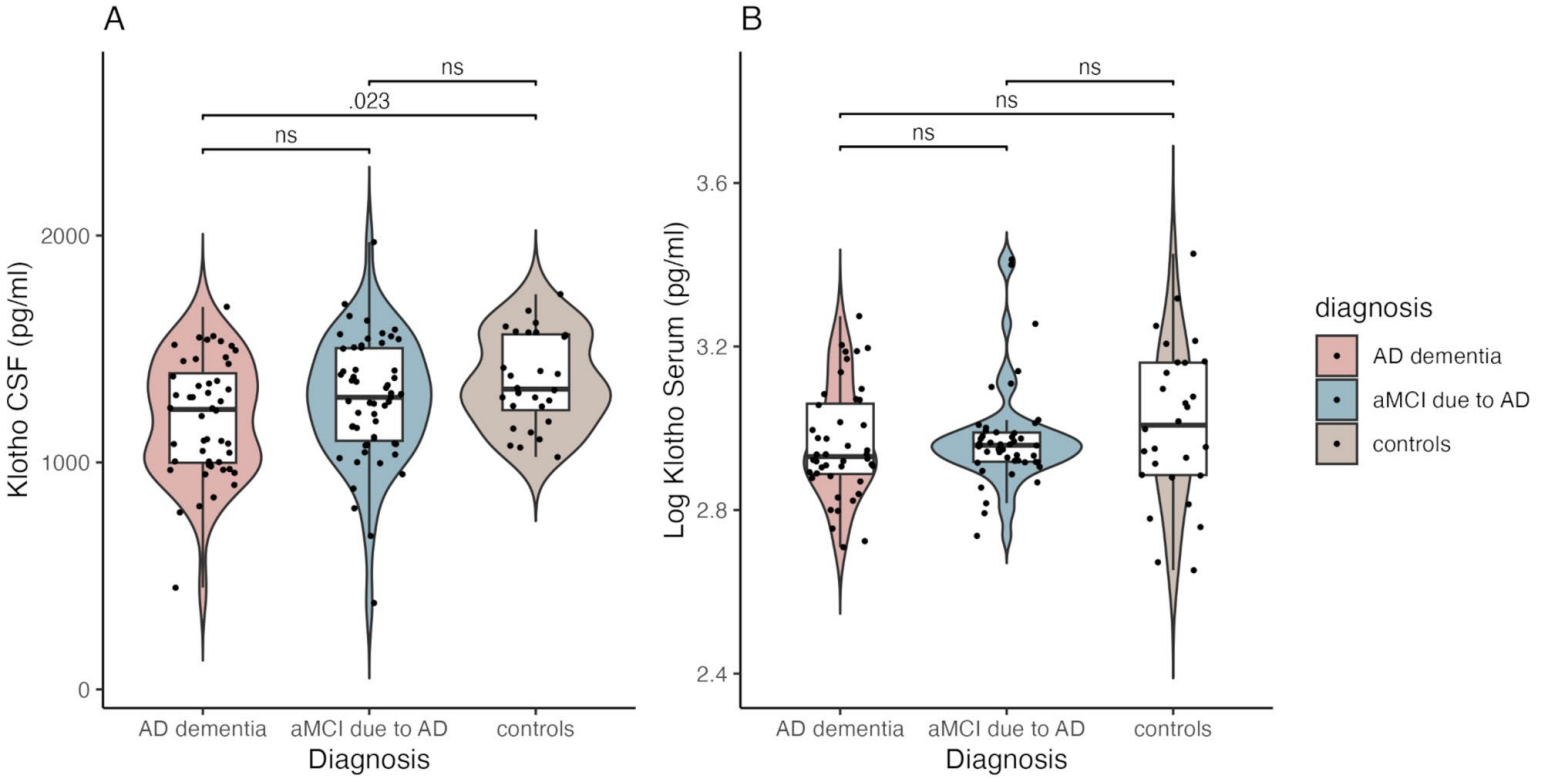
2 **A**) CSF sαKl concentrations in AD dementia, aMCI due to AD and controls. **2B**) Serum sαKl concentrations in AD dementia, aMCI due to AD and controls. Serum sαKl data are presented as log-transformed values. Exact *p*-values are displayed for significant group comparisons; “ns” = non-significant

The mean concentration of CSF sαKl was highest in the control group (1359.5 ± 208.1 pg/mL), followed by the aMCI due to AD group (1276.4 ± 227.0 pg/mL), and the AD-dementia group (1196.3 ± 262.4 pg/mL) (Table 2; Fig. 2). ANOVA revealed a significant effect of the study group on CSF sαKl levels (F[2,118] = 3.60, *p* = .030). Post-hoc test indicated that there was a significant difference between the AD dementia and control group (*p* = .028) but not between the control and aMCI due to AD group (*p* = .364) or between the aMCI due to AD and AD dementia group (*p* = .217). The significant main effect for the study group was explained away when we added age and sex into the analysis (F[2,118] = 1.31, *p* = .273).

With respect to serum sαKl levels, again, the control group had the highest average sαKl concentration (1129.1 ± 459.0 pg/mL), followed by the aMCI due to AD group (992.3 ± 392.6 pg/mL) and the AD dementia group (962.8 ± 316.9 pg/mL) (Table 2; Fig. 2). The main effect of study group in relation to serum sαKl concentration did not reach statistical significance (F[2, 118] = 1.79, *p* = .172).

### Association between sαKl levels and cognitive performance

In the unadjusted model, a modest positive relationship was observed between CSF sαKl levels and memory (β = 0.19, *p* = .025) in the entire sample. However, this relationship was attenuated and no longer statistically significant after adjusting the model for age, sex, and years of education (β = 0.08, *p* = .348). We observed a similar trend for language, which also showed a positive association with CSF sαKl levels (β = 0.24, *p* = .007), with the association no longer evident after adjusting the model for age, sex, and years of education (β = 0.08, *p* = .367). We also did not observe an association between CSF sαKl levels and language or memory performance when analyzing the study groups separately or when stratifying the analysis based on *APOE* ε4 status. Finally, there were no significant associations between CSF sαKl levels with any of the other cognitive domains we investigated after controlling for age, sex, and years of education (all *p* > .05).

We did not observe any significant associations between serum sαKl levels and memory or any of the other investigated cognitive domains (all *p* > .05), whether analyzing the entire sample, examining the diagnostic groups separately, or stratifying the analyses by *APOE* ε4 carrier status. The regression coefficients of each of the linear regression models showing CSF or serum based sαKl in relation to composite cognitive domain z-scores in the entire sample are presented in Supplementary Table 2, Additional File 2. The corresponding stratified models—by diagnostic group and *APOE* ε4 status—are reported in Supplementary Table 3, Additional File 3.

## Discussion

This study aimed to investigate *KL*-VSHET and sαKl protein levels in relation to diagnostic classification along the AD continuum and cognitive performance. First, we assessed whether *KL*-VSHET was associated with a reduced likelihood of aMCI or dementia due to AD compared to being cognitively intact and whether this association was different in *APOE* ε4 carriers compared to non-carriers. Second, we examined *KL*-VSHET’s influence on cognitive performance across the AD continuum, comparing the performance of *KL*-VSHET carriers and *KL*-VSNC. Third, we compared levels of serum and CSF sαKl protein in cognitively unimpaired controls, aMCI, and dementia due to AD individuals. Finally, we investigated whether CSF and serum sαKl levels were associated with better cognitive performance across the AD continuum. Through addressing these aims, our study contributes to the growing body of literature by examining domain-specific cognitive performance across biomarker-defined stages of the AD continuum, incorporating both CSF and serum sαKl levels as well as *KL*-VSHET haplotype. This integrated approach provides a more detailed view of how Klotho may relate to cognitive changes in AD.

We found that *KL*-VSHET carriers exhibited a 61% lower likelihood of being diagnosed with aMCI due to AD compared to being cognitively unimpaired and that this association was similar in both *APOE* ε4 carriers and non-carriers. Although the *p*-value only trended towards significance after controlling for all the covariates, including age, sex, and education (*p* = .061), the OR was 0.39, which corresponds to Cohen’s d of 0.52, a medium effect size. Post-hoc power estimates using G*Power software suggested that we had limited power (power of 0.66) to observe this result as statistically significant, hence predisposing our analyses towards Type II error bias. Further analysis revealed that among individuals with aMCI due to AD, *KL*-VSHET carriers were 48% less likely to be diagnosed with AD dementia, with a similar trend observed in both *APOE* ε4 carriers and non-carriers. Although this result did not reach statistical significance (*p* = .102), the observed effect size (Cohen’s d = 0.36) suggests a small-to-moderate association within the aMCI due to AD population. When examining the combined group of individuals with aMCI or dementia due to AD compared to cognitively unimpaired individuals, *KL*-VSHET carriers showed 41% lower odds of being classified within the cognitively impaired group (*p* = .205), with a stronger trend within *APOE* ε4 carriers. The OR of this result was 0.59, corresponding to a Cohen’s d of 0.29, a small effect size. Although none of these associations reached statistical significance individually, the consistency of the observed trends suggests that *KL*-VSHET may be associated with lower odds of aMCI or dementia due to AD, a notion that should be explored further in future research with larger sample sizes and a longitudinal follow-up.

Our results partially align with findings by Belloy and colleagues [11], who also reported a decreased risk of AD in individuals with the *KL*-VSHET haplotype, but only among *APOE* ε4 carriers, whereas we observed similar results for *APOE* ε4 carriers vs. non-carriers. Mechanistically, *KL*-VSHET might exert its protective effect by reducing the buildup of key AD proteins in the brain, Aβ and phosphorylated tau. Studies have shown lower age- and *APOE* ε4 -related Aβ and tau burden in cognitively unimpaired *KL*-VSHET carriers at risk for AD [10, 13, 15, 17, 45]. While some studies found no protective effect of *KL*-VSHET on Aβ and tau burden in *APOE* ε4 non-carriers [11, 12, 17], others did observe an effect in this subgroup [14, 15]. Our findings are consistent with the latter, suggesting that *KL*-VSHET might be associated with lower odds of aMCI and dementia due to AD in both *APOE* ε4 carriers and non-carriers. This potential spectrum of *KL*-VSHET influence across *APOE* ε4 status warrants further exploration with larger samples and more robust study designs to definitively disentangle the nuances of this interaction.

We also found that *KL*-VSHET carriers in the aMCI due to AD displayed significantly better memory compared to *KL*-VSNC, even after controlling for age, sex, and education, particularly when we restricted the analyses to *APOE* ε4 carriers. The same association was not evident in AD dementia individuals. These results may reflect a stage-specific effect of *KLOTHO*-related mechanisms. Prior studies suggest that *KL*-VSHET may be most effective during earlier phases of the disease, when amyloid and tau pathology are present but widespread neurodegeneration has not yet occurred [10, 14, 15]. In more advanced stages of AD, neurodegenerative changes may overwhelm any neuroprotective influence conferred by *KL*-VSHET. Together, these findings suggest that *KL*-VSHET may (a) help support memory function and (b) serve as a buffer against the deleterious effects of *APOE* ε4 on memory, specifically in the aMCI stage of AD.

Our results are consistent with those of Neitzel and colleagues, who also found that *KL*-VSHET was associated with better memory performance. They further noted that the observed association may have been explained by reduced tau burden facilitated by the presence of the effect of *KL*-VSHET haplotype, especially in *APOE* ε4 carriers [14].

*KL*-VSHET did not associate with other cognitive domains, implying its influence might be specific to memory. Previously, some have reported an association between *KL*-VSHET and better cognitive performance, both in healthy individuals and AD patients [6, 13, 16, 46]. Studies have shown positive associations with specific cognitive domains, such as executive function and memory [13, 46], but also broader global cognition [6, 16]. However, others have found no such association or even results where *KL*-VSHET reflected poorer cognitive performance [45, 47–51]. These inconsistencies potentially arise from the moderating influence of age and the complex interplay between *KL*-VSHET and other biological and epigenetic factors, which demand further investigation. Additionally, it is crucial to consider how *KL*-VSHET’s impact on cognition might differ within the specific context of pathological conditions like AD.

We found no significant differences in CSF or serum sαKl levels between the study groups. In the initial analysis, CSF sαKl levels appeared to differ across the study groups, with controls showing the highest levels, followed by the aMCI due to AD and AD dementia, and a statistically significant difference between controls and AD dementia, resembling the decline observed by Grøndvedt and colleagues [18]. However, while in our analyses, this association was reduced to non-significant after adjusting for age and sex, Grøndvedt et al.‘s findings remained significant even after adjustment. Semba and colleagues also found lower CSF sαKl in individuals with AD compared to cognitively unimpaired controls [22], supporting the notion of CSF-specific changes potentially missed by blood measurements. Yet one other study reported higher plasma sαKl l in AD patients, contrary to our serum sαKl findings [25]. Other studies observed no significant changes in plasma sαKl across AD stages [18, 24]. This alignment with our results suggests a potentially limited influence of AD pathology on serum and plasma sαKl levels.

With respect to CSF and serum sαKl in relation to memory and other cognitive domains, we found a tentative association with memory and language overall that was explained again by age, sex, and education. Analyzing individual study groups or stratifying by *APOE* ε4 status also showed no significant associations between CSF sαKl and memory or other cognitive domains. Similarly, serum sαKl levels lacked significant associations with memory or any other assessed cognitive domains. The weak positive correlation observed between CSF and serum sαKl levels suggests that while there is some degree of systemic-central coupling, sαKl in CSF vs. serum likely reflect distinct biological processes. While serum sαKl is predominantly derived from peripheral sources such as the kidney, CSF sαKl is believed to originate primarily from the choroid plexus, indicating central nervous system-specific production. This divergence may help explain why cognitive associations in our study appeared more pronounced for CSF sαKl compared to serum sαKl, although even the CSF-based findings were modest and did not remain statistically significant after adjustment for demographic variables. Together, these results suggest that while sαKl might be involved in AD pathology, its relationship with memory, at least as measured in this study, appears muted and possibly modulated by demographic factors. These findings contradict previous research where higher levels of sαKl among *KL*-VSHET carriers were used to explain the results between *KL*-VSHET and better memory [6]. While animal and human studies show cognitive benefits from elevated sαKl [20, 21, 52–55], our findings suggest this relationship might be more complex in individuals with AD.

### Limitations

This study has several limitations that should be considered. First, the cross-sectional study design does not allow for causal inferences to be drawn. Second, the sample size in this study was relatively small, causing limited statistical power and potentially restricting the generalizability of the findings. Post-hoc power estimates using G*Power software indicated that several comparisons had limited statistical power, including those examining differences in diagnostic classification odds between *KL*-VSHET and *KL*-VSNC (power of 0.66 for aMCI, 0.50 for AD dementia), as well as the subsequently stratified analyses by *APOE* ε4 carrier status (0.11–0.33), indicating a bias towards a Type II error. Therefore, null findings in our study may reflect this bias as much as a true lack of a statistically significant association. In the same context, we did not adjust our results for comparison-wise Type I error, which in our view would exacerbate the existing bias towards Type II error due to lower-than-ideal power. Additionally, *KL*-VS haplotype and sαKl protein data were not co-collected for all participants, limiting the ability to directly explore genotype-phenotype associations. While we did not assess the relationship between KL-VSHET or sαKl protein and AD biomarkers in this study, these associations were investigated in a prior publication [56]. Finally, although the ELISA kit used in this study demonstrated acceptable intra- and inter-assay variability, the precision of serum sαKl measurements at picogram concentrations may still be limited, given the inherently low abundance of the analyte and potential sensitivity constraints of immunoassay-based detection.

## Conclusions

As hypothesized, we observed that carriers of the *KL*-VSHET haplotype were substantially (although often non-significantly) underrepresented among participants with aMCI due to AD and AD dementia compared to cognitively unimpaired individuals, suggesting a potential association with lower likelihood of cognitive impairment. Contrary to our hypotheses, we found that the association between *KL*-VSHET and diagnostic group classification was similar for *APOE* ε4 carriers vs. non-carriers, as opposed to being prominent in *APOE* ε4 carriers only. In addition, we found that the carriers of *KL*-VSHET with aMCI due to AD displayed better memory performance compared to those without the haplotype, especially in combination with the risk-increasing *APOE* ε4, suggesting a buffering effect *KL*-VSHET against the established negative effect of *APOE* ε4 on memory. These findings deserve further attention, particularly within longitudinal research and with larger samples.

We did not find consistent evidence for an association between CSF or serum sαKl levels and cognitive status or memory performance in our sample. While previous studies reported associations between sαKl protein and AD pathology, our findings suggest that these relationships might be complex and influenced by demographic factors like age and sex, requiring further research with appropriate control and stratification strategies.

While our findings remain tentative due to limitations, they highlight the potential of *KL*-VSHET and sαKl protein factor as a target for further investigation and emphasize the need for larger, comprehensive studies. Future research should prioritize longitudinal designs, larger sample sizes, and detailed stratification based on genetic and demographic factors to provide more conclusive evidence.

## Supplementary Information

Below is the link to the electronic supplementary material.

Supplementary Material 1

Supplementary Material 2

Supplementary Material 3

## Data Availability

No datasets were generated or analysed during the current study.

## Abbreviations

aMCI: Amnestic mild cognitive impairment
ANCOVA: Analysis of covariance
ANOVA: Analysis of variance
APOE: Apolipoprotein E
Aβ: Amyloid beta
AD: Alzheimer’s disease
BNT: Boston Naming Test
CDT: Clock Drawing Test
CI: Confidence interval
CSF: Cerebrospinal fluid
CV: Coefficient of variance
DS-B: Digit Span Backward
DS-F: Digit Span Forward
ELISA: Enzyme-linked immunosorbent assay
GFR: Glomerular filtration rate
KL-VS: KLOTHO-VS haplotype
KL-VSHET: KLOTHO-VS heterozygosity
KL-VSNC: KLOTHO-VS non-carriers
LM-DR: Logical Memory Story I, delayed recall
LM-IR: Logical Memory Story I, immediate recall
MMSE: Mini-mental state examination
MRI: Magnetic resonance imaging
OR: Odds ratio
PET: Positron emission tomography
P-VF: Phonemic verbal fluency
ROCFT: Rey-Osterrieth Complex Figure Test
ROCFT-T: Rey-Osterrieth Complex Figure, copy condition
S-VF: Semantic verbal fluency
sαKl: Soluble α-klotho protein
TMT: Trail making test
